# Nonoptimal Codon Usage Is Critical for Protein Structure and Function of the Master General Amino Acid Control Regulator CPC-1

**DOI:** 10.1128/mBio.02605-20

**Published:** 2020-10-13

**Authors:** Xueliang Lyu, Yi Liu

**Affiliations:** a Department of Physiology, The University of Texas Southwestern Medical Center, Dallas, Texas, USA; b State Key Laboratory of Agricultural Microbiology, College of Plant Science and Technology, Huazhong Agricultural University, Wuhan, Hubei, China; Duke University

**Keywords:** codon usage, CPC-1, Neurospora, cross-pathway control, GCN4, cotranslational protein folding, translation elongation

## Abstract

Under amino acid starvation conditions, eukaryotic organisms activate a general amino acid control response. In Neurospora crassa, Cross Pathway Control Protein 1 (CPC-1), the ortholog of the Saccharomyces cerevisiae bZIP transcription factor GCN4, functions as the master regulator of the general amino acid control response. Codon usage biases are a universal feature of eukaryotic genomes and are critical for regulation of gene expression. Although codon usage has also been implicated in the regulation of protein structure and function, genetic evidence supporting this conclusion is very limited. Here, we show that *Neurospora cpc-1* has a nonoptimal NNU-rich codon usage profile that contrasts with the strong NNC codon preference in the genome. Although substitution of the *cpc-1* NNU codons with synonymous NNC codons elevated CPC-1 expression in *Neurospora*, it altered the CPC-1 degradation rate and abolished its amino acid starvation-induced protein stabilization. The codon-manipulated CPC-1 protein also exhibited different sensitivity to limited protease digestion. Furthermore, CPC-1 functions in rescuing the cell growth of the *cpc-1* deletion mutant and activation of the expression of its target genes were impaired by the synonymous codon changes. Together, these results reveal the critical role of codon usage in regulation of CPC-1 expression and function and establish a genetic example of the importance of codon usage in protein folding.

## Abstract (precis)

The general amino acid control response is critical for adaptation of organisms to amino acid starvation conditions. The preference to use certain synonymous codons is a universal feature of all genomes. Synonymous codon changes were previously thought to be silent mutations. In this study, we showed that the *Neurospora cpc-1* gene has an unusual codon usage profile compared to other genes in the genome. We found that codon optimization of the *cpc-1* gene without changing its amino acid sequence resulted in elevated CPC-1 expression, an altered protein degradation rate, and impaired protein functions due to changes in protein structure. Together, these results reveal the critical role of synonymous codon usage in regulation of CPC-1 expression and function and establish a genetic example of the importance of codon usage in protein structure.

## INTRODUCTION

Transcriptional regulation allows organisms to respond to changes in environmental conditions. Under amino acid starvation conditions, fungi activate a general amino acid control response that induces expression of genes involved in amino acid biosynthesis (1–4). The signal transduction pathways that mediate these responses are similar in eukaryotic cells from yeast to mammals. In the budding yeast Saccharomyces cerevisiae and the filamentous fungus Neurospora crassa, bZIP transcription factors GCN4 and Cross-Pathway Control Protein 1 (CPC-1), respectively, are the master transcriptional regulators that activate amino acid biosynthetic genes in response to amino acid limiting conditions (1–6). Like GCN4, CPC-1 binds to the 5′-TGA(C/G)TCA-3′ motifs in target gene promoters to activate transcription (1–3, 5).

A mechanism involving upstream open reading frames (uORFs) in *GCN4* modulates GCN4 protein production (1, 2). Under normal conditions, the translation of the uORFs prevents translation initiation from the *GCN4* ORF, resulting in the suppression of GCN4 expression. Under amino acid starvation conditions, however, the scanning 40S ribosomes bypass the uORFs and initiate translation at the downstream *GCN4* ORF, resulting in the induction of GCN4 protein expression. This type of uORF-mediated mechanism is conserved in general amino acid control responses from fungi to mammals: translational induction of CPC-1 in *Neurospora* and of ATF4 in mammals is controlled by this mechanism (7, 8). We recently showed that impaired tRNA I34 modification also triggers an amino acid starvation-like response (9). Ribosome profiling experiments, which were used to monitor ribosome occupancy on translating mRNAs, showed that there were many more ribosomes bypassing the two *cpc-1* uORFs and translating the downstream open reading frame region when tRNA I34 modification was suppressed (9).

Posttranslational regulation of GCN4 stability is another mechanism that contributes to its upregulation in response to amino acid starvation (1). GCN4 is very unstable when yeast cells are cultured in rich medium, with a half-life of several minutes, but its degradation becomes much slower under amino acid starvation conditions (10, 11). The degradation of GCN4 is mediated by the proteasome ubiquitination pathway and is dependent on its phosphorylation by cyclin-dependent kinases (11–13). It is not clear whether amino acid availability also regulates CPC-1 stability in *Neurospora*.

Due to the degeneracy of genetic code, most amino acids are encoded by two to six synonymous codons. Codon usage bias, the preference for certain synonymous codons for almost all amino acids, has been found in all genomes examined (14–17). Codon usage bias is an important determinant of gene expression levels in both eukaryotes and prokaryotes (18–21). We and other groups previously showed that codon usage regulates translation elongation speed: common codons enhance the local rate of translation elongation, whereas rare codons slow translation elongation (22–25). Rare codons preferentially cause ribosome stalling on an mRNA during translation, and this can result in premature translation termination and reduce translation efficiency (22, 24, 26). Furthermore, codon usage bias can regulate gene expression by affecting transcription (27–31).

In addition to the effect of codon usage on gene expression (32), accumulating biochemical and genetic evidence suggests that codon usage can also influence the cotranslational protein folding process through its effects on translation elongation speed, which influences the time available for cotranslational folding (19, 22, 26, 28, 33–46). It was previously shown in Escherichia coli that codon usage can affect the protein activity and structures of some overexpressed proteins (37, 38, 40, 43, 47). In eukaryotes, a synonymous single-nucleotide polymorphism of the human *MDR1* gene was previously shown to cause altered protein activity of the MDR1 protein transiently overexpressed in human cells, suggesting the involvement of codon usage in eukaryotic protein folding (39). More recently, codon usage was also shown to influence protein activity and/or structures of several other human proteins (28, 45, 48–50). However, those previous studies relied on protein overexpression, which could also influence protein folding in cells, and the degree of impact of codon usage on protein structure/function was often modest.

By studying the circadian clock genes in *Neurospora* and *Drosophila*, we previously demonstrated that the codon usage of circadian clock gene *frq* in *Neurospora* and *Per* in *Drosophila* plays a major role in determining the protein structure and function *in vivo* (19, 36). Importantly, those studies did not use protein overexpression and the functional impacts of codon usage on protein function and structure were very robust in these genetic systems, thus confirming the physiological role of codon usage in protein folding in eukaryotic systems. Furthermore, genome-wide correlations between gene codon usage and predicted protein structures have been observed in prokaryotes and eukaryotes, suggesting that codon usage functions as a universal code to broadly modulate protein folding (33, 51–53). However, there are currently only very few genetic examples that demonstrate the robust physiological influence of codon usage on protein folding and function (19, 36, 38).

N. crassa has a strong codon usage bias for C/G at wobble positions (33, 54), but we observed that the *cpc-1* gene has an abnormal NNU-rich codon usage bias. Amino acid starvation triggers the stabilization of yeast GCN4, and we observed a similar starvation-induced stabilization of CPC-1 protein. By changing the NNU codons of *cpc-1* to synonymous NNC codons, we demonstrated that the codon usage of *cpc-1* is required for CPC-1 stabilization in response to amino acid starvation, and that it is critical for the CPC-1 structure and function *in vivo*. Together, our results demonstrate the role of codon usage in controlling CPC-1 expression and function and establish another genetic example of the importance of codon usage in protein folding.

## RESULTS

### Abnormal codon usage profile of *cpc-1*.

**FIG 1:**
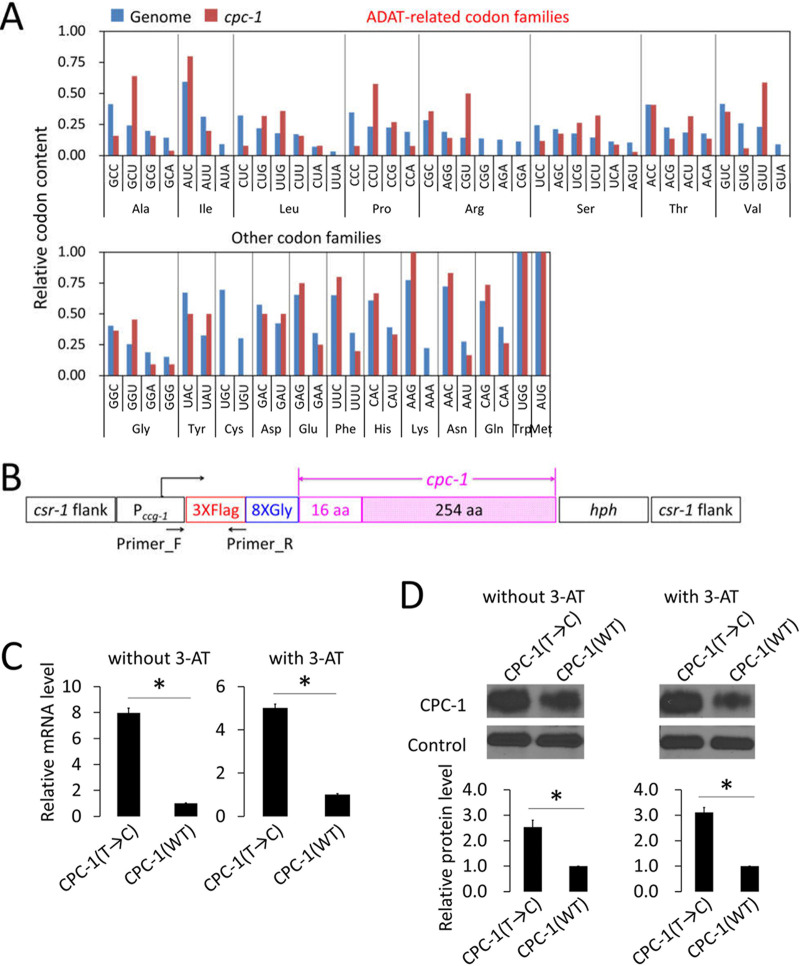
The ADAT-related NNU-rich codon usage of *cpc-1* contributes to the regulation of CPC-1 production. (A) The codon usage of *cpc-1* represented by the relative codon contents in each codon family compared to the genome-wide average codon usage. (B) Graphical representation of the constructs used for the expression of *cpc-1*(WT) and optimized *cpc-1*(T→C). To avoid the influences of the *cpc-1* uORFs and 5′ UTR, the *ccg-1* promoter (P*_ccg-1_*) and its 5′ UTR were used to drive the expression of *cpc-1*. The constructs were integrated into the *csr-1* locus in the N. crassa genome by homologous recombination. To avoid the influence of codon optimization on translation initiation, the first 50 codons (including the codons of 3×Flag and 8×Gly and 16 codons at the N terminus of *cpc-1*) were kept the same in both constructs. In *cpc-1*(T→C), 71 of the 270 ADAT-related NNU codons were changed to the most preferred NNC codons of N. crassa genome. For details, see Fig. S2. (C) The relative mRNA levels of *cpc-1*(WT) and *cpc-1*(T→C) detected by qRT-PCR in the host strain cultured in 2% glucose medium with or without 5 mM 3-aminotriazole (3-AT). The *cpc-1* mRNA levels were normalized to that of the *β-tubulin* gene (NCU04054). Primers used for qRT-PCR were designed to correlate to the 5′ region of the transcript, which is common to the two constructs (as shown in panel B) to ensure the same amplification efficiency. The *cpc-1*(WT) transcript level was set as 1.0. (D) (Upper panel) Western blot analysis of CPC-1 expressed in the *cpc-1*(WT) and *cpc-1*(T→C) strains cultured in 2% glucose medium with or without 5 mM 3-AT. A nonspecific constitutive band detected by the anti-Flag antibody was used as the control. (Lower panel) Densitometric analyses of the CPC-1 levels from three independent experiments. The CPC-1 protein level produced from *cpc-1*(WT) was set as 1.0. Data in panels C and D are means ± standard deviations (SD) (*n* = 3). *, *P* < 0.05, as determined by Student's two-tailed *t* test.

Examination of the N. crassa *cpc-1* gene revealed that it has an unusual codon usage profile. The *Neurospora* genome has a strong preference for NNC codons in every ADAT-related codon family (the A34 positions of their corresponding tRNAs can be converted to I34 by adenosine deaminases acting on tRNAs, known as ADATs) and for NNC/NNG codons in other codon families (33, 54). In contrast, for *cpc-1*, NNU codons are the most preferred codons for five (Ala, Pro, Arg, Ser, and Val) of the eight ADAT-related codon families (Fig. 1A). For Leu codons of *cpc-1*, the normally preferred CUC codon is one of the least used codons; it has a lower usage frequency than CUU. The usage frequency of ACU, which codes for Thr, is also higher in *cpc-1* than the genome average (Fig. 1A). Interestingly, the genome-preferred NNC codons are also not the preferred codons for the majority of the ADAT-related codon families in homologous *cpc-1* genes in Neurospora tetrasperma, Sordaria macrospora, and Aspergillus nidulans (see Fig. S1 in the supplemental material), suggesting that nonoptimal *cpc-1* codon usage is conserved. These results raised the possibility that the nonoptimal nature of the *cpc-1* codon usage profile is functionally important.

10.1128/mBio.02605-20.1 FIG S1 Codon usage profiles for ADAT-related codon families of *cpc-1* homologous genes in *Neurospora tetrasperma*, Sordaria macrospora, and Aspergillus nidulans. The relative codon contents compared to the genome-wide average codon usage in each codon family are shown. Download FIG S1, PDF file, 0.3 MB. Copyright © 2020 Lyu and Liu. 2020 Lyu and Liu. This content is distributed under the terms of the Creative Commons Attribution 4.0 International license.

10.1128/mBio.02605-20.2 FIG S2 Sequence alignment of the coding sequences of *cpc-1*(WT) and optimized *cpc-1*(T→C). Asterisks (*) indicate conserved sites. Download FIG S2, PDF file, 0.04 MB. Copyright © 2020 Lyu and Liu. 2020 Lyu and Liu. This content is distributed under the terms of the Creative Commons Attribution 4.0 International license.

### Regulation of CPC-1 expression by *cpc-1* codon usage.

The unusual NNU-rich codon usage profile of *cpc-1* suggests that it may play a biological role in regulating CPC-1 expression or function. To test this hypothesis, we created two versions of the *cpc-1* ORF: *cpc-1*(WT) (*cpc-1* wild type), in which all native codons were maintained, and *cpc-1*(T→C), in which all eight ADAT-related NNU codons (except for the codons for the N-terminal 16 amino acids) were substituted synonymously with the genome-preferred NNC codons without altering the amino acid sequence (Fig. S2). Thus, *cpc-1*(T→C) has more optimal codons than the wild-type (WT) *cpc-1* gene. We expressed 5′ epitope-tagged versions of *cpc-1*(WT) and *cpc-1*(T→C) ORFs under the control of the *ccg-1* promoter and the *ccg-1* 5′ untranslated region (5′ UTR) to exclude the effect of the uORFs and the 5′ UTR of *cpc-1* on translation. To minimize the potential impact of codon usage on translation initiation, the N-terminal regions (which include 3×Flag, an 8×Gly linker, and the codons for the initial 16 N-terminal amino acids of CPC-1) of the two versions of *cpc-1* were identical (Fig. 1B). Constructs containing the *cpc-1* transgenes were individually transformed into *Neurospora* strain 87-3 at the targeted *csr-1* locus. Homokaryotic transformant strains were cultured in 2% glucose medium with or without 3-aminotriazole (3-AT). 3-AT treatment results in amino acid starvation in *Neurospora* because it is a competitive inhibitor of the product of *his-3* gene, which is an enzyme required for histidine biosynthesis (6, 55–57). Because of the cross-pathway control in *Neurospora*, depletion of one amino acid leads to a general amino acid starvation response (3).

Gene codon optimization usually results in increased mRNA and protein levels in *Neurospora* (27, 58, 59). As expected, the mRNA levels of *cpc-1*(T→C) were significantly higher than those of *cpc-1*(WT) when the genes were expressed in the host strain cultured in 2% glucose medium with or without 3-AT (Fig. 1C). Similarly, the CPC-1 protein levels were also upregulated in the *cpc-1*(T→C) strain (Fig. 1D). These results suggest that the NNU-rich codon usage profile of *cpc-1* suppresses CPC-1 expression.

### Codon usage of *cpc-1* and culture conditions affect CPC-1 protein stability.

**FIG 2:**
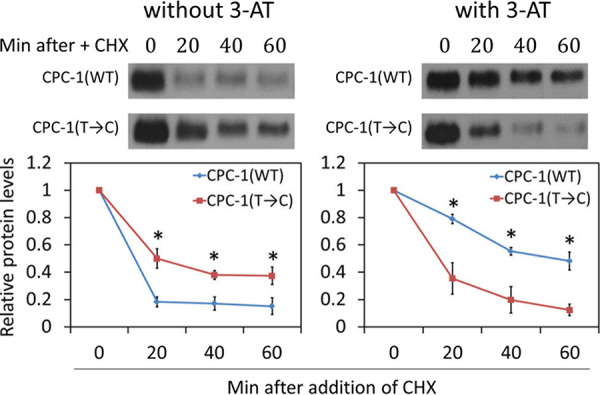
Codon usage optimization alters CPC-1 stability in response to amino acid starvation. (Upper panels) Representative Western blots showing the CPC-1 protein levels in *cpc-1*(WT) and *cpc-1*(T→C) strains grown in 2% glucose medium with or without 5 mM 3-AT. Cycloheximide (CHX, 10 μg ml^−1^) was added at time zero, and cultures were harvested at the indicated time points. (Lower panels) Densitometric analyses of the Western blot experiments described in the upper panels. Data are means ± SD (*n* = 3). *, *P* < 0.05, as determined by Student's two-tailed *t* test. Min, minutes.

GCN4, the ortholog of CPC-1 in S. cerevisiae, is rapidly degraded under rich nutrient conditions but is stabilized under amino acid starvation conditions, a response that contributes to GCN4 upregulation after amino acid starvation (1, 10, 11, 60). To determine whether CPC-1 protein stability is affected by amino acid starvation and codon usage, we compared the CPC-1 turnover rates measured after the addition of the protein synthesis inhibitor cycloheximide (CHX) in the *cpc-1*(WT) and *cpc-1*(T→C) strains grown in 2% glucose medium with and without 3-AT. 3-AT treatment resulted in marked stabilization of CPC-1 in the *cpc-1*(WT) strain (Fig. 2), suggesting that as, with GCN4 in yeast (1), protein stability was altered under amino acid starvation conditions. However, CPC-1 was more stable in the *cpc-1*(T→C) strain than in the *cpc-1*(WT) strain when the strains were grown in 2% glucose medium without 3-AT. Furthermore, the stabilization of CPC-1 observed in the *cpc-1*(WT) strain upon 3-AT treatment was not observed in the *cpc-1*(T→C) strain (Fig. 2). These results indicate that the NNU-biased *cpc-1* codon usage plays an important role in regulating CPC-1 protein stability under amino acid starvation conditions.

### *cpc-1* codon usage affects CPC-1 structure and function.

**FIG 3:**
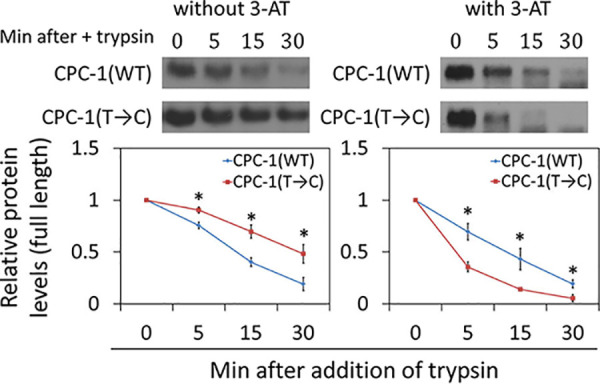
Codon usage affects CPC-1 cotranslational folding. (Top) Western blots of CPC-1 expression in the *cpc-1*(WT) and *cpc-1*(T→C) strains cultured in 2% glucose medium in the absence or presence of 5 mM 3-AT. Trypsin (0.25 μg/ml) was added into the freshly isolated protein extracts, and protein samples were analyzed at the indicated time points. (Bottom) Densitometric analyses of the full-length CPC-1 levels from the experiments described above. Data are means ± SD (*n* = 3). *, *P* < 0.05, as determined by Student's two-tailed *t* test.

The observed effect of *cpc-1* codon usage on CPC-1 protein stability raised the possibility that proper cotranslational folding of CPC-1 depends on codon usage as observed for other *Neurospora* proteins (19, 22, 24). To examine this possibility, we performed a limited trypsin digestion assay to probe the structure differences of CPC-1 proteins in the *cpc-1*(WT) and *cpc-1*(T→C) strains. The freshly isolated protein extracts of the *cpc-1*(WT) and *cpc-1*(T→C) strains were treated with trypsin, and the levels of full-length CPC-1 were determined by Western blot analyses as a function of digestion time. When the cultures were grown in 2% glucose medium without 3-AT, CPC-1 isolated from the *cpc-1*(T→C) strain was significantly more resistant to trypsin digestion than that isolated from the *cpc-1*(WT) strain, but it was more sensitive to trypsin digestion after 3-AT treatment (Fig. 3). These results indicate that codon usage influences CPC-1 protein structure.

Note that the *ccg-1* promoter-driven *cpc-1* expression did not result in its overexpression. In fact, we found that the *cpc-1* mRNA level under the control of the *ccg-1* promoter and 5′ UTR in a *cpc-1* knockout strain (*cpc-1Δ*) was actually much lower than the endogenous *cpc-1* level in a WT strain (Fig. S3). Thus, the effect of codon usage on CPC-1 structure is not due to its overexpression.

10.1128/mBio.02605-20.3 FIG S3 The relative *cpc-1* mRNA levels detected in the indicated strains. The relative expression levels of *cpc-1* were quantified in RPKM (reads per kilobase per million) values. Only reads mapped to the coding DNA sequence (CDS) region of *cpc-1* were taken into account. Download FIG S3, PDF file, 0.4 MB. Copyright © 2020 Lyu and Liu. 2020 Lyu and Liu. This content is distributed under the terms of the Creative Commons Attribution 4.0 International license.

**FIG 4:**
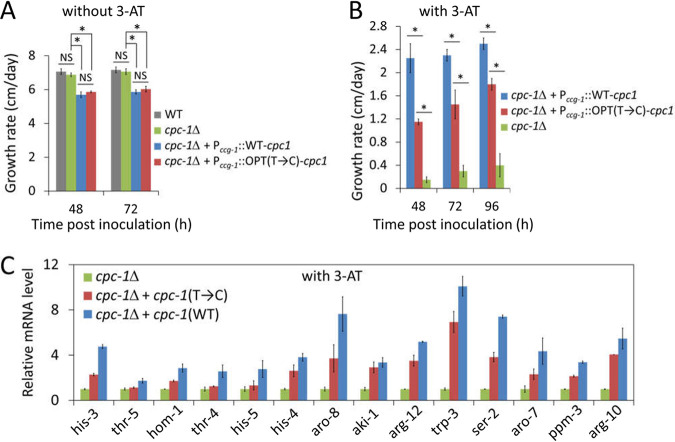
Optimization of *cpc-1* codon usage impairs CPC-1 biological functions. (A) Growth rates of the WT strain, the *cpc-1Δ* strain, and the *cpc-1Δ* strains expressing *cpc-1*(WT) or *cpc-1*(C→T) after 48 and 72 h as determined in race tube assays performed without 3-AT. *, *P* < 0.05; NS, not significant; as determined by Student's two-tailed *t* test. (B) Growth rates of the *cpc-1Δ* strain and the *cpc-1Δ* strains expressing *cpc-1*(WT) or *cpc-1*(C→T) after 48, 72, and 96 h as determined in race tube assays performed with 5 mM 3-AT. (C) The relative mRNA levels of selected CPC-1 target genes in the indicated strains. The relative mRNA level of each gene was determined by qRT-PCR, and the expression levels of the genes were normalized to that of the *β-tubulin* gene (NCU04054). The mRNA level of each gene in the *cpc-1Δ* strain was set as 1.0. Data in panels are means ± SD (*n* = 3).

To determine whether the structural differences caused by codon usage result in changes in protein function, we introduced the *cpc-1*(WT) and *cpc-1*(T→C) constructs individually into the *cpc-1Δ* strain. We then compared the abilities of these two constructs to rescue the growth defect of the *cpc-1Δ* mutant under amino acid starvation conditions. Under normal growth conditions, the WT and *cpc-1Δ* strains had similar growth rates but the *cpc-1Δ* strains expressing the *cpc-1*(WT) or *cpc-1*(T→C) had a slightly but significantly lower growth rate in constant light at room temperature (Fig. 4A). In constant light, *cpc-1*(WT) and *cpc-1*(T→C) are constitutively expressed, and *cpc-1* translation is not regulated by the uORFs due to the use of the *ccg-1* 5′ UTR in the transgene strains. The reduced growth rate in these strains is consistent with the known role of GCN4 as a repressor of protein synthesis (61). In the presence of 3-AT, the growth rate of the *cpc-1Δ* strain was dramatically reduced in a race tube assay (Fig. 4B). The growth phenotype was drastically improved in the *cpc-1*(WT) strain, indicating a functional rescue of the *cpc-1Δ* strain by the *cpc-1*(WT) transgene. The growth rate of the *cpc-1Δ*, *cpc-1*(T→C) strain, however, was much lower than that of the *cpc-1Δ*, *cpc-1*(WT) strain in the presence of 3-AT (Fig. 4B), indicating that the T→C codon usage profile changes impaired the CPC-1 protein function even though they increased the CPC-1 protein level (Fig. 1D).

To further confirm this conclusion, we compared the mRNA levels of 14 genes regulated by CPC-1 that are involved in amino acid biosynthesis (3, 9). The mRNA levels of these CPC-1 target genes in the *cpc-1Δ*; *cpc-1Δ*, *cpc-1*(WT)*Δ*; and *cpc-1Δ*, *cpc-1*(T→C) strains treated with 3-AT were determined. The mRNA levels of all the 14 genes were dramatically upregulated in the *cpc-1Δ*, *cpc-1*(WT) strain compared to those in the *cpc-1Δ* strain (Fig. 4C). As expected, the mRNA level induction of these genes was reduced in the *cpc-1Δ*, *cpc-1*(T→C) strain (Fig. 4C). Together, these results demonstrate that the NNU-biased codon usage profile of *cpc-1* plays an important role in determining CPC-1 protein structure and function *in vivo*.

## DISCUSSION

CPC-1 is the master transcription regulator of gene expression in *Neurospora* in response to amino acid starvation (3, 7, 55, 62). Like the situation of its yeast ortholog GCN4, expression of CPC-1 is also translationally regulated by a mechanism involving uORFs (7, 9). The translation of *cpc-1* can be translationally activated by bypassing its uORFs under amino acid starvation conditions. As reported previously for GCN4 (10, 11), here, we showed that 3-AT treatment triggers CPC-1 stabilization, which also contributes to CPC-1 accumulation under amino acid starvation conditions. Although the mechanism of CPC-1 degradation is not known, it is possible that, as with GCN4, amino acid starvation regulates posttranslational modification of CPC-1, which affects its degradation by the ubiquitin-proteasome pathway (11–13).

In this study, we demonstrated that the nonoptimal codon usage profile of *cpc-1* has a major impact on the structure and function of CPC-1. By changing the *cpc-1* codon usage from the NNU-rich profile to the NNC-rich profile typical of the *Neurospora* genome, we showed that codon usage is critical for CPC-1 protein structure and function. This conclusion was supported by several lines of evidence. First, the codon manipulation altered the CPC-1 protein degradation rate and abolished amino acid starvation-induced CPC-1 stabilization (Fig. 2), suggesting that codon usage can affect CPC-1 structure. Second, the codon optimization altered the sensitivity of CPC-1 to limited trypsin digestion, indicating that codon optimization affected protein structure (Fig. 3). Third, in the presence of 3-AT, CPC-1(T→C) was less stable and more sensitive to trypsin digestion than CPC-1 (WT) was (Fig. 2; see also Fig. 3), suggesting that codon usage-mediated structure changes of CPC-1 affected its ability to be regulated by potential posttranslational mechanisms triggered by amino acid starvation conditions. Fourth, despite the upregulation of CPC-1 protein levels in *Neurospora* upon codon optimization, expression of *cpc-1*(T→C) did not rescue the growth defects of the *cpc-1Δ* strain under amino acid starvation conditions as effectively as that of *cpc-1*(WT) did. Finally, the impaired CPC-1 function of the *cpc-1*(T→C) strain was further indicated by the reduced induction of CPC-1 target genes in response to amino acid starvation. Note that codon optimization of *cpc-1* did not cause its overexpression (see Fig. S3 in the supplemental material). Thus, our study established a critical physiological role of codon usage in regulating CPC-1 structure and function.

In addition, our analyses established another *in vivo* example of the influence of codon usage on protein structure. Due to the role of codon usage in regulating the rate of translation elongation, codon usage was previously proposed to influence the cotranslational protein folding process (19, 22, 26, 28, 33–38). However, genetic evidence in support of such a role of codon usage is quite limited. By studying the codon usage function of the circadian clock genes *frequency* in *Neurospora* and *Period* in *Drosophila*, we previously showed that codon usage plays an important role in affecting the structures and, therefore, the functions of these two proteins *in vivo* (19, 36). Similarly to the *frequency* and *Period* genes, *cpc-1* is enriched in nonoptimal codons. Additionally, as with FRQ and PER proteins, most regions of the CPC-1 protein are predicted to be intrinsically disordered. Our findings are consistent with the hypothesis that the cotranslational protein folding process is sensitive to codon usage-mediated translation elongation kinetics and that this process is regulated to ensure proper functioning of the proteins with intrinsically disordered domains. Further supporting this, we and others previously showed that nonoptimal codon usage correlated with predicted unstructured domains in a genome-wide manner in *Neurospora* and other organisms (33, 52). The structure of the DNA binding domain of the yeast GCN4 was previously shown to be flexible (63–65). GCN4 exhibits a concentration-dependent α-helical transition: the transition of the GCN4 basic region from an unfolded to a folded conformation depends on its accessibility to DNA binding sites (65). Such properties may make it more sensitive to the cotranslational folding process.

Taken together, our results suggest that the unusual codon profile of *cpc-1* represents another example of evolutionary adaption that results in its optimal protein structure and function in response to environmental changes.

## MATERIALS AND METHODS

### Strains and growth conditions.

N. crassa strain 87-3 (*bd*, *a*) was used as the control and was further used as the host strain for the expression of various versions of *cpc-1* unless otherwise specified. For the growth rate assay, the FGSC 4200 (*a*, WT) strain was used as the control. The *cpc-1Δ* strain was obtained from the *Neurospora* knockout library (66). Liquid cultures were grown in 2% glucose medium (1× Vogel’s, 2% glucose) or in 0.1% glucose medium (1× Vogel’s, 0.1% glucose, 0.17% arginine). Race tube medium contained 1× Vogel’s, 0.1% glucose, 0.17% arginine, 50 ng ml^−1^ biotin, and 1.5% agar. All the strains were cultured on slants containing 1× Vogel’s, 2% sucrose, and 1.5% agar before various experiments were performed. All the strains were cultured under constant light at room temperature.

### Plasmid constructs.

For gene expression at the *csr-1* locus in N. crassa, a hygromycin B resistance gene (*hph*) was inserted downstream of the *ccg-1* promoter of a parental plasmid, Pcsr1, to create a new plasmid, Pcsr1-hyg. Pcsr1-hyg is a *csr-1*-targeting expression vector with an expression cassette in which P_ccg-1_ and *hph* flank the gene of interest, and this cassette is flanked by two *csr-1*-related fragments that serve as the double recombination sites (67). When this plasmid was transformed into N. crassa cells, it was integrated into the *csr-1* gene locus by replacing *csr-1* with the expression cassette by double homologous recombination. The resulting transformants were screened for both hygromycin B (200 μg ml^−1^) resistance and cyclosporine (5 μg ml^−1^) resistance conferred by the presence of *hph* and the absence of *csr-1*, respectively. The levels of efficiency and accuracy of this approach were very high (>90% positive transformants). In this study, two versions of *cpc-1*(WT) and *cpc-1*(T→C) with a 3×Flag tag and an 8×Gly linker at the N termini were separately introduced into the Pcsr1-hyg construct. The resulting constructs were transformed into host strains by electroporation. Homokaryon strains were obtained by microconidium purification.

### Protein stability and limited trypsin digestion assays.

For protein stability assay, the cycloheximide (CHX) working concentration and experimental procedures were the same as those previously described (19). For culture conditions, fresh conidia (1 week postinoculation on slants) of the host strains were cultured in 50 ml 2% glucose medium in plates at room temperature for 2 days. The cultures were cut into small discs with a diameter of 1 cm, and then the discs were transferred into flasks with the same liquid medium and were grown with orbital shaking (200 rpm) for one more day before addition of CHX (final concentration, 10 μg ml^−1^). For the samples treated with 3-AT, the culture discs in 2% glucose medium were treated with 5 mM 3-AT for 8 h before sample collection. Cells were collected at the indicated time points after addition of CHX. For the limited trypsin digestion assay, the culture conditions and sample collection procedures were the same as those described above except for the addition of CHX. The working concentration of trypsin was 0.25 μg/ml. Protein extraction and Western blot analyses were performed as previously described (68). Equal amounts of total proteins (100 μg) were loaded into all lanes of 7.5% SDS-PAGE gels containing 37.5:1 acrylamide/bisacrylamide. The primary and secondary antibodies used for detecting the 3×Flag were monoclonal anti-Flag M2 antibody produced in mouse (Sigma-Aldrich, catalog no. F3165) and goat anti-mouse IgG (H+L)-horseradish peroxidase (HRP) conjugate (Bio-Rad, catalog no. 170-6516), respectively. Densitometry was performed using Image J.

### Quantitative reverse transcription-PCR (qRT-PCR) and mRNA-seq.

For qRT-PCR, the sample collection procedures were the same as those described for the protein stability assay except that CHX was not added. For cultures treated with 3-AT as indicated in the figures, the liquid cultures were treated with 5 mM 3-AT for 8 h before sample collection. RNA extraction and qRT-PCR were performed as previously described (69). *β-tubulin* (NCU04054) was quantified as an internal control. Primers used for qRT-PCR are listed in Table S1 in the supplemental material. The relative mRNA levels of *cpc-1* in the WT, *cpc-1Δ*, and *cpc-1Δ*, *cpc-1*(WT) strains under amino acid starvation conditions were measured by determination of their RPKM (reads per kilobase per million) values from our high-throughput mRNA sequencing (mRNA-seq) data. The mRNA sequencing libraries used in this study were generated from cultures maintained in 2% glucose medium with 5 mM 3-AT treatment for 8 h before sample collection. The sample collection procedures were the same as those described for the protein stability assay except that CHX was not added. Total RNAs were extracted using TRIzol reagents (Invitrogen) and treated with DNase (Turbo DNase; Ambion). The libraries were prepared using NEBNext Ultra kits for RNA and sequenced by an Illumina HiSeq 2000 instrument. mRNA-seq experiments were performed by Joint Genome Institute (JGI) on an Illumina NovaSeq platform.

10.1128/mBio.02605-20.4 TABLE S1 Sequences of primers used in this study. Download Table S1, PDF file, 0.04 MB. Copyright © 2020 Lyu and Liu. 2020 Lyu and Liu. This content is distributed under the terms of the Creative Commons Attribution 4.0 International license.

### Codon manipulation and data collection from databases.

The codons of *cpc-1* were optimized based on N. crassa codon usage frequency data from the Codon Usage Database (https://www.kazusa.or.jp/codon/cgi-bin/showcodon.cgi?species=5141). The mutated sites for the optimized *cpc-1*(T→C) are shown in Fig. S2 in the supplemental material.

### Data availability.

The raw and processed sequencing data have been submitted to the NCBI Gene Expression Omnibus under accession number GSE150287.

## References

[1] Hinnebusch AG 2005 Translational regulation of *GCN4* and the general amino acid control of yeast. Annu Rev Microbiol 59:407–450. doi:10.1146/annurev.micro.59.031805.133833. 16153175

[2] Hinnebusch AG, Natarajan K 2002 Gcn4p, a master regulator of gene expression, is controlled at multiple levels by diverse signals of starvation and stress. Eukaryot Cell 1:22–32. doi:10.1128/ec.01.1.22-32.2002. 12455968 PMC118051

[3] Tian C, Kasuga T, Sachs MS, Glass NL 2007 Transcriptional profiling of cross pathway control in *Neurospora crassa* and comparative analysis of the Gcn4 and CPC1 regulons. Eukaryot Cell 6:1018–1029. doi:10.1128/EC.00078-07. 17449655 PMC1951524

[4] Sachs MS 1996 General and cross-pathway controls of amino acid biosynthesis, p 315–345. *In* Brambl R, Marzluf GA (ed), The Mycota: biochemistry and molecular biology, vol III Springer-Verlag, Heidelberg, Germany.

[5] Paluh JL, Yanofsky C 1991 Characterization of *Neurospora* CPC1, a bZIP DNA-binding protein that does not require aligned heptad leucines for dimerization. Mol Cell Biol 11:935–944. doi:10.1128/mcb.11.2.935. 1824960 PMC359753

[6] Ebbole DJ, Paluh JL, Plamann M, Sachs MS, Yanofsky C 1991 *cpc-1*, the general regulatory gene for genes of amino acid biosynthesis in *Neurospora crassa*, is differentially expressed during the asexual life cycle. Mol Cell Biol 11:928–934. doi:10.1128/mcb.11.2.928. 1824959 PMC359752

[7] Ivanov IP, Wei J, Caster SZ, Smith KM, Michel AM, Zhang Y, Firth AE, Freitag M, Dunlap JC, Bell-Pedersen D, Atkins JF, Sachs MS 2017 Translation initiation from conserved non-AUG codons provides additional layers of regulation and coding capacity. mBio 8:e00844-17. doi:10.1128/mBio.00844-17. 28655822 PMC5487733

[8] Vattem KM, Wek RC 2004 Reinitiation involving upstream ORFs regulates *ATF4* mRNA translation in mammalian cells. Proc Natl Acad Sci U S A 101:11269–11274. doi:10.1073/pnas.0400541101. 15277680 PMC509193

[9] Lyu X, Yang Q, Li L, Dang Y, Zhou Z, Chen S, Liu Y 2020 Adaptation of codon usage to tRNA I34 modification controls translation kinetics and proteome landscape. PLoS Genet 16:e1008836. doi:10.1371/journal.pgen.1008836. 32479508 PMC7289440

[10] Irniger S, Braus GH 2003 Controlling transcription by destruction: the regulation of yeast Gcn4p stability. Curr Genet 44:8–18. doi:10.1007/s00294-003-0422-3. 14508604

[11] Meimoun A, Holtzman T, Weissman Z, McBride HJ, Stillman DJ, Fink GR, Kornitzer D 2000 Degradation of the transcription factor Gcn4 requires the kinase Pho85 and the SCF(CDC4) ubiquitin-ligase complex. Mol Biol Cell 11:915–927. doi:10.1091/mbc.11.3.915. 10712509 PMC14820

[12] Shemer R, Meimoun A, Holtzman T, Kornitzer D 2002 Regulation of the transcription factor Gcn4 by Pho85 cyclin PCL5. Mol Cell Biol 22:5395–5404. doi:10.1128/mcb.22.15.5395-5404.2002. 12101234 PMC133946

[13] Chi Y, Huddleston MJ, Zhang X, Young RA, Annan RS, Carr SA, Deshaies RJ 2001 Negative regulation of Gcn4 and Msn2 transcription factors by Srb10 cyclin-dependent kinase. Genes Dev 15:1078–1092. doi:10.1101/gad.867501. 11331604 PMC312682

[14] Ikemura T 1985 Codon usage and tRNA content in unicellular and multicellular organisms. Mol Biol Evol 2:13–34. doi:10.1093/oxfordjournals.molbev.a040335. 3916708

[15] Sharp PM, Tuohy TM, Mosurski KR 1986 Codon usage in yeast: cluster analysis clearly differentiates highly and lowly expressed genes. Nucleic Acids Res 14:5125–5143. doi:10.1093/nar/14.13.5125. 3526280 PMC311530

[16] Comeron JM 2004 Selective and mutational patterns associated with gene expression in humans: influences on synonymous composition and intron presence. Genetics 167:1293–1304. doi:10.1534/genetics.104.026351. 15280243 PMC1470943

[17] Plotkin JB, Kudla G 2011 Synonymous but not the same: the causes and consequences of codon bias. Nat Rev Genet 12:32–42. doi:10.1038/nrg2899. 21102527 PMC3074964

[18] Xu Y, Ma P, Shah P, Rokas A, Liu Y, Johnson CH 2013 Non-optimal codon usage is a mechanism to achieve circadian clock conditionality. Nature 495:116–120. doi:10.1038/nature11942. 23417065 PMC3593822

[19] Zhou M, Guo J, Cha J, Chae M, Chen S, Barral JM, Sachs MS, Liu Y 2013 Non-optimal codon usage affects expression, structure and function of clock protein FRQ. Nature 495:111–115. doi:10.1038/nature11833. 23417067 PMC3629845

[20] Hense W, Anderson N, Hutter S, Stephan W, Parsch J, Carlini DB 2010 Experimentally increased codon bias in the *Drosophila Adh* gene leads to an increase in larval, but not adult, alcohol dehydrogenase activity. Genetics 184:547–555. doi:10.1534/genetics.109.111294. 19966063 PMC2828731

[21] Lampson BL, Pershing NL, Prinz JA, Lacsina JR, Marzluff WF, Nicchitta CV, MacAlpine DM, Counter CM 2013 Rare codons regulate KRas oncogenesis. Curr Biol 23:70–75. doi:10.1016/j.cub.2012.11.031. 23246410 PMC3567844

[22] Yu CH, Dang Y, Zhou Z, Wu C, Zhao F, Sachs MS, Liu Y 2015 Codon usage influences the local rate of translation elongation to regulate co-translational protein folding. Mol Cell 59:744–754. doi:10.1016/j.molcel.2015.07.018. 26321254 PMC4561030

[23] Weinberg DE, Shah P, Eichhorn SW, Hussmann JA, Plotkin JB, Bartel DP 2016 Improved ribosome-footprint and mRNA measurements provide insights into dynamics and regulation of yeast translation. Cell Rep 14:1787–1799. doi:10.1016/j.celrep.2016.01.043. 26876183 PMC4767672

[24] Yang Q, Yu CH, Zhao F, Dang Y, Wu C, Xie P, Sachs MS, Liu Y 2019 eRF1 mediates codon usage effects on mRNA translation efficiency through premature termination at rare codons. Nucleic Acids Res 47:9243–9258. doi:10.1093/nar/gkz710. 31410471 PMC6755126

[25] Hussmann JA, Patchett S, Johnson A, Sawyer S, Press WH 2015 Understanding biases in ribosome profiling experiments reveals signatures of translation dynamics in yeast. PLoS Genet 11:e1005732. doi:10.1371/journal.pgen.1005732. 26656907 PMC4684354

[26] Zhao F, Yu CH, Liu Y 2017 Codon usage regulates protein structure and function by affecting translation elongation speed in *Drosophila* cells. Nucleic Acids Res 45:8484–8492. doi:10.1093/nar/gkx501. 28582582 PMC5737824

[27] Zhou Z, Dang Y, Zhou M, Li L, Yu CH, Fu J, Chen S, Liu Y 2016 Codon usage is an important determinant of gene expression levels largely through its effects on transcription. Proc Natl Acad Sci U S A 113:E6117–E6125. doi:10.1073/pnas.1606724113. 27671647 PMC5068308

[28] Fu J, Dang Y, Counter C, Liu Y 2018 Codon usage regulates human KRAS expression at both transcriptional and translational levels. J Biol Chem 293:17929–17940. doi:10.1074/jbc.RA118.004908. 30275015 PMC6240855

[29] Kudla G, Lipinski L, Caffin F, Helwak A, Zylicz M 2006 High guanine and cytosine content increases mRNA levels in mammalian cells. PLoS Biol 4:e180. doi:10.1371/journal.pbio.0040180. 16700628 PMC1463026

[30] Newman ZR, Young JM, Ingolia NT, Barton GM 2016 Differences in codon bias and GC content contribute to the balanced expression of TLR7 and TLR9. Proc Natl Acad Sci U S A 113:E1362–E1371. doi:10.1073/pnas.1518976113. 26903634 PMC4791032

[31] Zhou Z, Dang Y, Zhou M, Yuan H, Liu Y 2018 Codon usage biases co-evolve with transcription termination machinery to suppress premature cleavage and polyadenylation. Elife 7:e33569. doi:10.7554/eLife.33569. 29547124 PMC5869017

[32] Quax TE, Claassens NJ, Söll D, van der Oost J 2015 Codon bias as a means to fine-tune gene expression. Mol Cell 59:149–161. doi:10.1016/j.molcel.2015.05.035. 26186290 PMC4794256

[33] Zhou M, Wang T, Fu J, Xiao G, Liu Y 2015 Nonoptimal codon usage influences protein structure in intrinsically disordered regions. Mol Microbiol 97:974–987. doi:10.1111/mmi.13079. 26032251 PMC4636118

[34] Chaney JL, Clark PL 2015 Roles for synonymous codon usage in protein biogenesis. Annu Rev Biophys 44:143–166. doi:10.1146/annurev-biophys-060414-034333. 25747594

[35] Komar AA 2009 A pause for thought along the co-translational folding pathway. Trends Biochem Sci 34:16–24. doi:10.1016/j.tibs.2008.10.002. 18996013

[36] Fu J, Murphy KA, Zhou M, Li YH, Lam VH, Tabuloc CA, Chiu JC, Liu Y 2016 Codon usage affects the structure and function of the *Drosophila* circadian clock protein PERIOD. Genes Dev 30:1761–1775. doi:10.1101/gad.281030.116. 27542830 PMC5002980

[37] Sander IM, Chaney JL, Clark PL 2014 Expanding Anfinsen's principle: contributions of synonymous codon selection to rational protein design. J Am Chem Soc 136:858–861. doi:10.1021/ja411302m. 24392935 PMC3959793

[38] Walsh IM, Bowman MA, Soto Santarriaga IF, Rodriguez A, Clark PL 2020 Synonymous codon substitutions perturb cotranslational protein folding in vivo and impair cell fitness. Proc Natl Acad Sci U S A 117:3528–3534. doi:10.1073/pnas.1907126117. 32015130 PMC7035613

[39] Kimchi-Sarfaty C, Oh JM, Kim IW, Sauna ZE, Calcagno AM, Ambudkar SV, Gottesman MM 2007 A “silent” polymorphism in the *MDR1* gene changes substrate specificity. Science 315:525–528. doi:10.1126/science.1135308. 17185560

[40] Buhr F, Jha S, Thommen M, Mittelstaet J, Kutz F, Schwalbe H, Rodnina MV, Komar AA 2016 Synonymous codons direct cotranslational folding toward different protein conformations. Mol Cell 61:341–351. doi:10.1016/j.molcel.2016.01.008. 26849192 PMC4745992

[41] O'Brien EP, Ciryam P, Vendruscolo M, Dobson CM 2014 Understanding the influence of codon translation rates on cotranslational protein folding. Acc Chem Res 47:1536–1544. doi:10.1021/ar5000117. 24784899

[42] Zhang G, Ignatova Z 2011 Folding at the birth of the nascent chain: coordinating translation with co-translational folding. Curr Opin Struct Biol 21:25–31. doi:10.1016/j.sbi.2010.10.008. 21111607

[43] Zhang G, Hubalewska M, Ignatova Z 2009 Transient ribosomal attenuation coordinates protein synthesis and co-translational folding. Nat Struct Mol Biol 16:274–280. doi:10.1038/nsmb.1554. 19198590

[44] Pechmann S, Chartron JW, Frydman J 2014 Local slowdown of translation by nonoptimal codons promotes nascent-chain recognition by SRP in vivo. Nat Struct Mol Biol 21:1100–1105. doi:10.1038/nsmb.2919. 25420103 PMC4488850

[45] Kim SJ, Yoon JS, Shishido H, Yang Z, Rooney LA, Barral JM, Skach WR 2015 Translational tuning optimizes nascent protein folding in cells. Science 348:444–448. doi:10.1126/science.aaa3974. 25908822

[46] Sauna ZE, Kimchi-Sarfaty C 2011 Understanding the contribution of synonymous mutations to human disease. Nat Rev Genet 12:683–691. doi:10.1038/nrg3051. 21878961

[47] Komar AA, Lesnik T, Reiss C 1999 Synonymous codon substitutions affect ribosome traffic and protein folding during in vitro translation. FEBS Lett 462:387–391. doi:10.1016/s0014-5793(99)01566-5. 10622731

[48] Kirchner S, Cai Z, Rauscher R, Kastelic N, Anding M, Czech A, Kleizen B, Ostedgaard LS, Braakman I, Sheppard DN, Ignatova Z 2017 Alteration of protein function by a silent polymorphism linked to tRNA abundance. PLoS Biol 15:e2000779. doi:10.1371/journal.pbio.2000779. 28510592 PMC5433685

[49] Alexaki A, Hettiarachchi GK, Athey JC, Katneni UK, Simhadri V, Hamasaki-Katagiri N, Nanavaty P, Lin B, Takeda K, Freedberg D, Monroe D, McGill JR, Peters R, Kames JM, Holcomb DD, Hunt RC, Sauna ZE, Gelinas A, Janjic N, DiCuccio M, Bar H, Komar AA, Kimchi-Sarfaty C 2019 Effects of codon optimization on coagulation factor IX translation and structure: implications for protein and gene therapies. Sci Rep 9:15449. doi:10.1038/s41598-019-51984-2. 31664102 PMC6820528

[50] Hunt R, Hettiarachchi G, Katneni U, Hernandez N, Holcomb D, Kames J, Alnifaidy R, Lin B, Hamasaki-Katagiri N, Wesley A, Kafri T, Morris C, Bouche L, Panico M, Schiller T, Ibla J, Bar H, Ismail A, Morris H, Komar A, Kimchi-Sarfaty C 2019 A single synonymous variant (c.354G>A [p.P118P]) in *ADAMTS13* confers enhanced specific activity. Int J Mol Sci 20:5734. doi:10.3390/ijms20225734. PMC6888508 31731663

[51] Thanaraj TA, Argos P 1996 Protein secondary structural types are differentially coded on messenger RNA. Protein Sci 5:1973–1983. doi:10.1002/pro.5560051003. 8897597 PMC2143259

[52] Pechmann S, Frydman J 2013 Evolutionary conservation of codon optimality reveals hidden signatures of cotranslational folding. Nat Struct Mol Biol 20:237–243. doi:10.1038/nsmb.2466. 23262490 PMC3565066

[53] Clarke TF IV, Clark PL 2010 Increased incidence of rare codon clusters at 5' and 3' gene termini: implications for function. BMC Genom 11:118. doi:10.1186/1471-2164-11-118. PMC2833160 20167116

[54] Radford A, Parish JH 1997 The genome and genes of *Neurospora crassa*. Fungal Genet Biol 21:258–266. doi:10.1006/fgbi.1997.0979. 9290240

[55] Sachs MS, Yanofsky C 1991 Developmental expression of genes involved in conidiation and amino acid biosynthesis in *Neurospora crassa*. Dev Biol 148:117–128. doi:10.1016/0012-1606(91)90322-t. 1834495

[56] Brennan MB, Struhl K 1980 Mechanisms of increasing expression of a yeast gene in *Escherichia coli*. J Mol Biol 136:333–338. doi:10.1016/0022-2836(80)90377-0. 6990004

[57] Joung JK, Ramm EI, Pabo CO 2000 A bacterial two-hybrid selection system for studying protein–DNA and protein–protein interactions. Proc Natl Acad Sci U S A 97:7382–7387. doi:10.1073/pnas.110149297. 10852947 PMC16554

[58] Frumkin I, Lajoie MJ, Gregg CJ, Hornung G, Church GM, Pilpel Y 2018 Codon usage of highly expressed genes affects proteome-wide translation efficiency. Proc Natl Acad Sci U S A 115:E4940–E4949. doi:10.1073/pnas.1719375115. 29735666 PMC6003480

[59] Jeacock L, Faria J, Horn D 2018 Codon usage bias controls mRNA and protein abundance in trypanosomatids. Elife 7:e32496. doi:10.7554/eLife.32496. 29543155 PMC5896881

[60] Streckfuss-Bomeke K, Schulze F, Herzog B, Scholz E, Braus GH 2009 Degradation of *Saccharomyces cerevisiae* transcription factor Gcn4 requires a C-terminal nuclear localization signal in the cyclin Pcl5. Eukaryot Cell 8:496–510. doi:10.1128/EC.00324-08. 19218424 PMC2669204

[61] Mittal N, Guimaraes JC, Gross T, Schmidt A, Vina-Vilaseca A, Nedialkova DD, Aeschimann F, Leidel SA, Spang A, Zavolan M 2017 The Gcn4 transcription factor reduces protein synthesis capacity and extends yeast lifespan. Nat Commun 8:457. doi:10.1038/s41467-017-00539-y. 28878244 PMC5587724

[62] Paluh JL, Orbach MJ, Legerton TL, Yanofsky C 1988 The cross-pathway control gene of *Neurospora crassa*, *cpc-1*, encodes a protein similar to GCN4 of yeast and the DNA-binding domain of the oncogene v-*jun*-encoded protein. Proc Natl Acad Sci U S A 85:3728–3732. doi:10.1073/pnas.85.11.3728. 2967496 PMC280291

[63] Thompson KS, Vinson CR, Freire E 1993 Thermodynamic characterization of the structural stability of the coiled-coil region of the bZIP transcription factor GCN4. Biochemistry 32:5491–5496. doi:10.1021/bi00072a001. 8504069

[64] Saudek V, Pastore A, Castiglione Morelli MA, Frank R, Gausepohl H, Gibson T, Weih F, Roesch P 1990 Solution structure of the DNA-binding domain of the yeast transcriptional activator protein GCN4. Protein Eng 4:3–10. doi:10.1093/protein/4.1.3. 2290831

[65] Weiss MA, Ellenberger T, Wobbe CR, Lee JP, Harrison SC, Struhl K 1990 Folding transition in the DMA-binding domain of GCN4 on specific binding to DNA. Nature 347:575–578. doi:10.1038/347575a0. 2145515

[66] Colot HV, Park G, Turner GE, Ringelberg C, Crew CM, Litvinkova L, Weiss RL, Borkovich KA, Dunlap JC 2006 A high-throughput gene knockout procedure for *Neurospora* reveals functions for multiple transcription factors. Proc Natl Acad Sci U S A 103:10352–10357. doi:10.1073/pnas.0601456103. 16801547 PMC1482798

[67] Bardiya N, Shiu PK 2007 Cyclosporin A-resistance based gene placement system for *Neurospora crassa*. Fungal Genet Biol 44:307–314. doi:10.1016/j.fgb.2006.12.011. 17320431

[68] Garceau NY, Liu Y, Loros JJ, Dunlap JC 1997 Alternative initiation of translation and time-specific phosphorylation yield multiple forms of the essential clock protein FREQUENCY. Cell 89:469–476. doi:10.1016/s0092-8674(00)80227-5. 9150146

[69] Xue Z, Ye Q, Anson SR, Yang J, Xiao G, Kowbel D, Glass NL, Crosthwaite SK, Liu Y 2014 Transcriptional interference by antisense RNA is required for circadian clock function. Nature 514:650–653. doi:10.1038/nature13671. 25132551 PMC4214883

