# Supplementary figures and images for: Nonoptimal Codon Usage Is Critical for Protein Structure and Function of the Master General Amino Acid Control Regulator CPC-1
Source: mBio. 2020 Oct 13;11(5):e02605-20. doi: 10.1128/mBio.02605-20 (PMC7554675; doi:10.1128/mBio.02605-20)

Figure S1

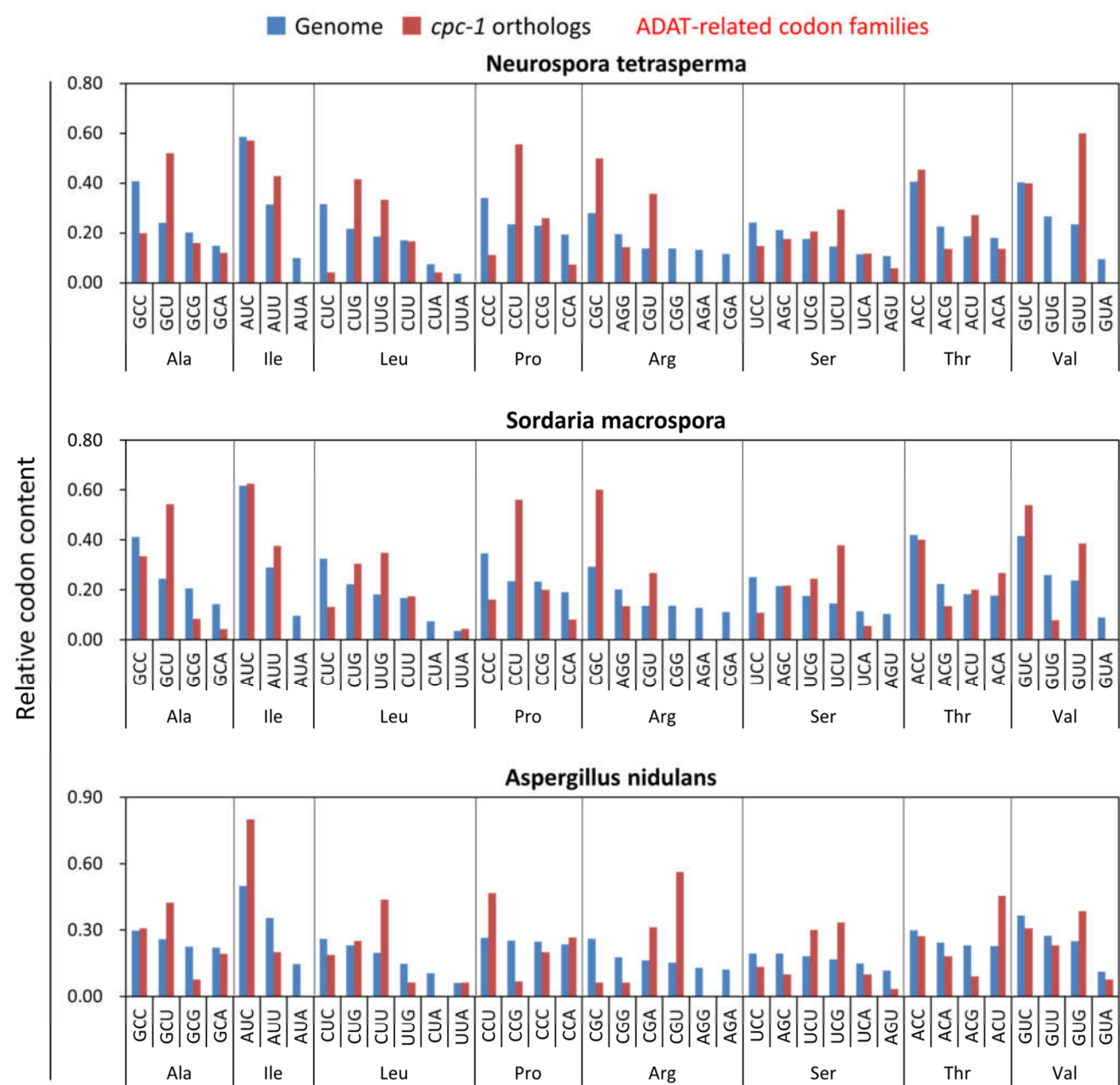

Supplement: FIG S1 [file mBio.02605-20-sf001.pdf]

Figure S3

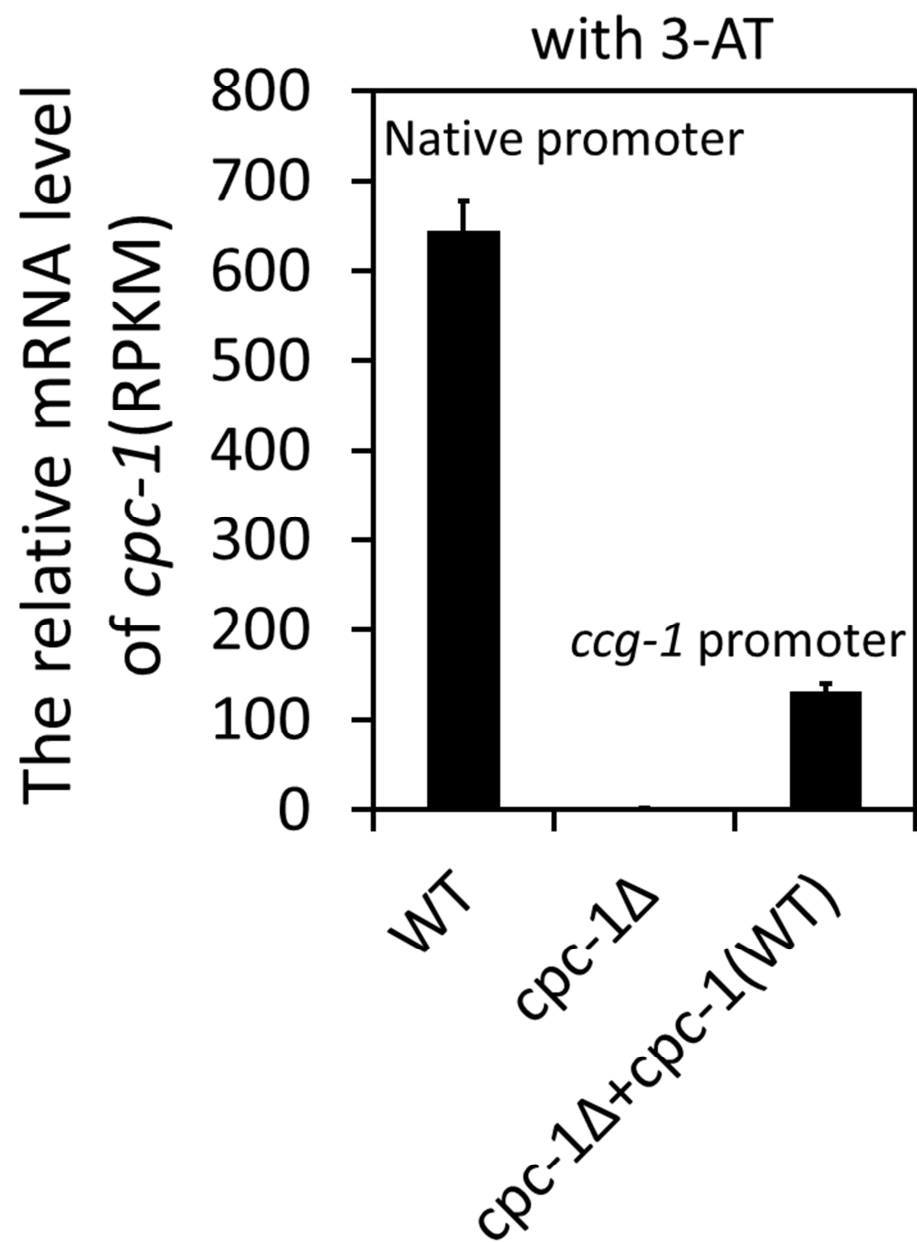

Supplement: FIG S3 [file mBio.02605-20-sf003.pdf]
