# Supplementary material for: Nonoptimal Codon Usage Is Critical for Protein Structure and Function of the Master General Amino Acid Control Regulator CPC-1
Source: mBio. 2020 Oct 13;11(5):e02605-20. doi: 10.1128/mBio.02605-20 (PMC7554675; doi:10.1128/mBio.02605-20)
Supplement: FIG S2 [file mBio.02605-20-sf002.pdf]

Figure S2

```
cpc-1(WT)     ATGTTCTCAGAACTGGACCTGCTGGATTTCGCCACCTTTGACGGTGGAGCTACCACCGAGGCGGCGTTCGCTTCGCCTGCCAACCAGACATACGATCTTTCGAGCAGCGTTCCTAGCTCC
cpc-1(T→C)    ATGTTCTCAGAACTGGACCTGCTGGATTTCGCCACCTTTGACGGTGGAGCCACCACCGAGGCGGCGTTCGCCTCGCCCGCCAACCAGACATACGATCTCTCGAGCAGCGTCCCCAGCTCC
              ************************************************** ******************** ***** ******************** *********** ** ******

cpc-1(WT)     GTCTCCAACATGGGCACTGTCTCGCCTCAGGAGCTTCTGCTCCATGAGCCGTATCTGTCTGCTCCTAGCTCCACTGCTCTTACGGCTTTGACTTCGCCTTCGCTCTTCGACGGCTCACCG
cpc-1(T→C)    GTCTCCAACATGGGCACCGTCTCGCCCCAGGAGCTCCTGCTCCATGAGCCGTATCTGTCCGCCCCCAGCTCCACCGCCCTCACGGCCTTGACCTCGCCCTCGCTCTTCGACGGCTCACCG
              ***************** ******** ******** *********************** ** ** ******** ** ** ***** ***** ***** *********************

cpc-1(WT)     GATTTCGATACCTTCGACATCTCTCCTAATTTTGGGCACAGTGACCTGGAAAACCCAGACACGTGGTTCTCACTTTTCCCTGATGCCACCCCGCTACCTCAAGCTCAGGCTCAGGTCCAG
cpc-1(T→C)    GATTTCGATACCTTCGACATCTCCCCCAATTTTGGGCACAGTGACCTGGAAAACCCAGACACGTGGTTCTCACTCTTCCCCGATGCCACCCCGCTACCCCAAGCCCAGGCCCAGGTCCAG
              *********************** ** *********************************************** ***** ***************** ***** ***** *********

cpc-1(WT)     ACTCAGCCTCAGACCCAGACTCAGACTGAGCAGCAAACACAACCGCTGCCTGAGTTGGTTCAATCTGTGCAGCCGACCGTTCAGCCGACCGTTGAGCAAACAGTTCACTCTGTTGAAGCT
cpc-1(T→C)    ACCCAGCCCCAGACCCAGACCCAGACCGAGCAGCAAACACAACCGCTGCCCGAGTTGGTCCAATCCGTGCAGCCGACCGTCCAGCCGACCGTCGAGCAAACAGTCCACTCCGTCGAAGCC
              ** ***** *********** ***** *********************** ******** ***** ************** *********** *********** ***** ** *****

cpc-1(WT)     TCGCCTGCTACCCCATCTGAGGATTTGGAGGTTTTGTCTCCGGGCTCTGGTCATCAGAGGAGGAAGTCGTCTGTCAGCCCTCCCAGCGGTCGTCACTCGTCGGTCGCTGGTGTTGGCTCT
cpc-1(T→C)    TCGCCCGCCACCCCATCCGAGGATTTGGAGGTCTTGTCCCCGGGCTCCGGTCATCAGAGGAGGAAGTCGTCCGTCAGCCCCCCCAGCGGTCGCCACTCGTCGGTCGCCGGTGTCGGCTCC
              ***** ** ******** ************** **** ********** *********************** ******** *********** ************** ***** *****

cpc-1(WT)     CGCCGTCGTGACAAGCCTCTACCCCCTATCATTGTTGAGGATCCTTCCGATGTCGTTGCCATGAAGCGTGCTCGCAACACTTTGGCTGCTCGCAAGTCTCGTGAGCGCAAGGCTCAGCGC
cpc-1(T→C)    CGCCGCCGCGACAAGCCCCTACCCCCCATCATCGTCGAGGATCCCTCCGATGTCGTCGCCATGAAGCGCGCCCGCAACACCTTGGCCGCCCGCAAGTCCCGCGAGCGCAAGGCCCAGCGC
              ***** ** ******** ******** ***** ** ******** *********** *********** ** ******** ***** ** ******** ** *********** ******

cpc-1(WT)     TTGGAGGAGTTGGAAGCTAAGATCGAGGAACTGATCGCAGAGCGTGACCGTTGGAAGAACTTGGCTTTGGCGCACGGTGCGTCTACGGAGTAA
cpc-1(T→C)    TTGGAGGAGTTGGAAGCCAAGATCGAGGAACTGATCGCAGAGCGCGACCGCTGGAAGAACTTGGCCTTGGCGCACGGTGCGTCCACGGAGTAA
              ***************** ************************** ***** ************** ***************** *********
```
