# Supplementary material for: Nonoptimal Codon Usage Is Critical for Protein Structure and Function of the Master General Amino Acid Control Regulator CPC-1
Source: mBio. 2020 Oct 13;11(5):e02605-20. doi: 10.1128/mBio.02605-20 (PMC7554675; doi:10.1128/mBio.02605-20)
Supplement: TABLE S1 [file mBio.02605-20-st001.pdf]

Table S1. Primers used for RT-PCR.

Gene	Forward primer (5'-3')	Reverse primer (5'-3')
cpc-1(5'UTR)	CTTCTTCCATCATCAGCCAACA	CCTTGTCATCGTCATCCTTGTAG
his-3	CCTCGTTCGTCAAGCACATTA	CTCCTCAACCTTAGCCAACTG
thr-5	GATTGGCGGTGGACTAAGAC	TGGTGTCAACAGGATGAAGAAG
hom-1	GGATACACTGTCAGCGTTGG	TCAGCGTTAAGGATGGAGGAA
thr-4	CGTGCGGATGTTACCTTCAA	CGTCATGGACTCGACAATCTT
his-5	AAGGTGATGCGTGCGAACAA	GTCTCGTTGCTCGGCTTTCC
his-4	GCGTCGGCTACAGAACTATC	GTGCTTCAGCGAGGTAAGAA
aro-8	ATGTCGCTTGTCCTCCTCTG	ACTTCCTTGCCGTTGATGGT
aki-1	CCGAGATGAAGAACATGATTGGTAT	CAGGAAGGTGAACAAGTTGGTAT
arg-12	GTCCTCAATGCTTCTGCTTACAA	TGCTTCGCTTGCTGAACATC
trp-3	ACCTATATCCTTCAGAACCAATACG	GCTCGGTATCCTTCCAGTTG
ser-2	GCTGCTAACGGTGACTACTT	GGTGAGGATGATGTTGTTGAG
aro-7	GGAGAAGGAGAAGCACAAGGA	GGTGAGGTCAACGAACAACTG
ppm-3	CGCAAGATTAGCAACCACCTA	CATCCACGATCCTCAATCCATT
arg-10	CTGGCTGTCAAGAAGGAGTG	GCTCATTGGCAGTGTGGATA
β-tubulin	GCGTATCGGCGAGCAGTT	CCTCACCAGTGTACCAATGCA
